# Changes in forward light scatter parameters as a function of refractive error in young adults

**DOI:** 10.1007/s00417-019-04584-9

**Published:** 2020-01-07

**Authors:** Manbir Nagra, Mansi Patel, John Barbur

**Affiliations:** 1 School of Health and Care Professions, University of Portsmouth, Portsmouth, PO1 2DT UK; 2 Applied Vision Research Centre, Division of Optometry and Visual Science, City, University of London, London, EC1V 0HB UK

**Keywords:** Refractive error, Forward light scatter, Retinal straylight, Myopia, Axial length

## Abstract

**Background/aims:**

Some aspects of visual performance worsen with increasing myopia. Whilst the underlying causes are not always clear, reduction in retinal image quality is often attributed to structural changes in the posterior myopic eye. Forward light scatter, originating principally from the cornea and lens, is known to produce veiling glare which subsequently reduces retinal image contrast. It is therefore of interest to investigate whether forward light scatter varies with refractive error.

**Methods:**

Thirteen young-adult subjects (18–25 years), with mean spherical errors (MSE ± sd, D) RE, − 1.69 ± 2.02 (range 0.38 to − 4.75); LE, − 1.91 ± 1.94 (range 0.50 to − 4.63) underwent binocular assessment of forward light scatter using the AVOT light scatter test. Five glare annuli, with effective eccentricities ranging from 2 to 10°, were used to estimate parameters, *k* and *n*, which define the light scatter function of the eye. These were then used to calculate the area under the light scatter function (*k*′) and the total volume of light scatter (*k*″).

**Results:**

Significant correlation was found between increasing myopia and *k*′ values (RE, *p* < 0.05; *r* = 0.64; LE, *p* < 0.05, *r* = 0.66). Neither the ‘volume’ of light scatter (*k*″), the parameter, *n*, which controls the angular distribution of light scatter, or the straylight parameter constant, *k*, were significantly correlated with refractive error (*p* > 0.05 for both eyes). Axial length was also not correlated with any of the light scatter parameters measured.

**Conclusion:**

The preliminary data from this study provide evidence that some light scatter parameters may be correlated with refractive error. Further studies are needed to characterize how changes in the anterior media of the eye, and inclusion of a wider range of refractive errors, may affect forward light scatter.

## Introduction

Some visual functions in myopes are often poorer when compared with emmetropes [1–3], even when refractive errors are fully corrected. Owing to the strong association between increasing myopia and axial length elongation, the impaired visual response is frequently attributed to structural and neural changes relating to the posterior region of the myopic eye. It has been speculated that reduced retinal function in myopia may include loss of cell function [4]; reduced retinal cell density [5]; and misalignment of photoreceptors [6, 7].

A less well studied cause of retinal image degradation is forward light scatter, also known as retinal straylight. Forward light scatter originates predominantly in the cornea (approximately 30%) [8] and the crystalline lens [9]. The forward light scatter within the retina is believed to be negligible by comparison. The backscatter from the retina and, in particular, the deeper structures involving the retinal pigment epithelium can be quite significant in older individuals, but this light is less useful in decreasing the effective retinal image contrast because of the directional sensitivity of cones. Significant deviations from normal cone alignment towards the centre of the pupil have, however, been reported in myopia [10].

Forward light scatter produces a veil on the retina, which in turn contributes most to reduced retinal image contrast. Scattered light gives rise to ‘disability glare’, haloes [11], in the case of bright single light sources, and can adversely affect many visual tasks, particularly those requiring the detection of fine spatial detail. In this sense, changes in retinal image as a result of forward light scatter in the eye are equivalent to low pass spatial filtering with higher spatial frequencies being affected most.

Any structural changes which disturb the normal functioning of the cornea and lens can cause increased scattered light, for example, early stage cataract, corneal oedema and refractive surgery [10, 12, 13]. Whilst the effects of scattered light on retinal image contrast can be significant, Snellen acuity (measured with close to 100% contrast optotypes) remains relatively unaffected and explains why scattered light is often overlooked during routine clinical examination [14].

Only a handful of studies have investigated the link between forward light scatter and refractive error; the findings of which have been inconsistent. Rozema et al. [15] used the single ring, C-Quant straylight metre (Oculus Optikgeräte, Wetzlar, Germany) to measure straylight parameters in a large cohort (*n* = 518 eyes, mean participant age ± sd, 39.7 ± 13.2, range 8.5 to 78 years) with reference to axial length (mean ± sd, 23.9 ± 1.3 mm, range 19.85–28.70) and mean spherical refractive error (mean ± sd, − 1.50 ± 2.90, range − 10.75 to 8.4 D). The study reported a significant adverse effect of both increasing axial length and myopic refractive error on retinal straylight. More recently, Guber et al. [16] measured retinal straylight using the C-Quant on the dominant eye of 45 young adults (mean age ± sd, 33.13 ± 10.25 years; range, 21 to 59 years) with an average mean spherical error of − 1.26 ± 1.80D. Refractive errors were categorized as emmetropia (*n* = 14); myopia (*n* = 16); hyperopia (*n* = 8) and astigmatism (*n* = 7). Specifically, myopic error ranged from − 1.25 D to − 5.25 D. Whilst Guber et al. did not find a statistically significant effect of refractive error on straylight, the authors do, however, note an observed reduction in straylight in myopes compared with emmetropes, although the effect failed to reach statistical significance. The Optical Quality Analysis System (OQAS), which is based on the double-pass technique, has also been used to estimate intraocular light scatter with respect to refractive error [17, 18]. Whilst Miao et al. [18] noted an increase in objective scatter index with increasing myopia, Martínez-Roda et al. [17] did not; a finding which was, in part, attributed to refractive error differences between cohorts.

In this study, we aim to investigate the effects of refractive error and axial length on a range of forward light scatter parameters. The experiments were carried out using the City University, Light Scatter (LS) test (City Occupational Ltd., London, UK) which employs five extended annuli as light scatter sources and uses a flicker-nulling method, similar to that described by Van Den Berg and Spekreijse [19] to measure both the amount and angular distribution of scattered light in the eye [20].

The LS test allows complete assessment of light scatter parameters, including the integral of the light scatter function and the ‘volume’ of scattered light expected from a point source. Other tests of light scatter employ a single ring of fixed angular subtense and calculate the same parameters by assuming that the angular distribution of scattered light remains unchanged and equal to that expected for the normal eye.

## Materials and methods

The study was approved by the Optometry departmental proportionate ethics review committee of City University of London. All aspects of the investigation were conducted in line with the tenets of the Declaration of Helsinki. Informed consent was obtained from all individual participants included in the study.

Young-adult participants (18–25 years) were recruited from a university staff-student body. Thirteen participants fulfilled the selection criteria and were included in the study. Insufficient information was available on the magnitude of the effects and the expected variability to carry out any justifiable power calculation to assess the minimum sample size. It was therefore decided to examine as many subjects as possible within the restricted time available in the hope that the expected effects would be sufficiently robust, even when only a small number of subjects could be examined within the time available for the project. High astigmatism (≥ 1.50 DC) and individuals with any signs of ocular pathologies were not included in the study. Objective measurements of non-cycloplegic refractive error were obtained using the WAM-5500 open-view binocular autorefractor (Ryusyo Industrial Co. Ltd., Osaka, Japan). Measurements were expressed as mean spherical error (MSE, in D). Axial length (AL) was measured using the Topcon Aladdin biometer (Topcon, UK).

Each participant carried out assessment of forward light scatter using the City University, LS test; habitual refractive corrections, where appropriate, were worn throughout. The LS test includes a ‘learning’ task which each participant undertakes prior to the experiment. A dark disc of 0.8° diameter is always present at the centre of each glare source together with a glare source annulus of varying size. The stimulus consists of a glare source burst of 7.5-Hz rapid flicker that lasts for ~ 400 ms. The test employs the flicker-nulling technique to measure two zero-flicker thresholds for each annulus. Interleaved staircases are employed, and the participant’s task is to report when flicker is no longer detected. The first threshold represents the luminance modulation depth of the dark disc that is just below that caused by scattered light. The second threshold corresponds to a disc modulation amplitude that is higher than that caused by scattered light when the participant also just fails to detect rapid flicker. The difference between the two thresholds is a measure of the participant’s sensitivity to flicker for the stimulus conditions employed in the LS test. The midpoint between the two thresholds is taken as a best estimate of the luminance of the external source that generates the same retinal illuminance as the glare source. When all the measurements are completed and the veiling luminance estimated for each annulus, the program computes the best parameters, *k*, and, *n*, in the light scatter equation, L_S_/E = k · θ^−n^, to fit the measured data. *E* represents the illuminance level generated by the glare source (in lumens/m^2^) in the plane of the pupil which is known for each annulus; *k* is the eye specific, light scatter parameter; and *n* is the scatter index which determines the angular distribution of forward light scatter in the eye [21].

Numerical methods are used in the LS test to compute the area under the light scatter function (i.e. parameter *k*′) from 2 to 90° as well as the volume of ‘scatted light’ (expected from 2 to 90°) one obtains when rotating the line scatter function through 360° (i.e. parameter *k*″). The program then displays the fitted light scatter function and the computed parameters (for graphical output of the results see LS test: https://www.city.ac.uk/avot).

### Statistical analyses

Statistical analyses were conducted using IBM SPSS Statistics (IBM UK Ltd. Portsmouth, UK). 0.05 was taken as the level of statistical significance was for all comparisons. Linear regression fits were used to test for significant correlations between each of the light scatter parameters (*k*′, *k*″, *n*) and AL and MSE. Given the strong correlation between AL and MSE, partial correlations were used to control for this covariance. Since light scatter functions were tested binocularly, data for both eyes are shown.

## Results

Mean spherical errors were (MSE ± sd, D) − 1.69 ± 2.02 (range 0.38 to − 4.75) for the right eye and − 1.91 ± 1.94 (range 0.50 to − 4.63) for the left eye with anisometropia ≤ 1.25 D. Mean axial lengths (mm ± sd) were 24.24 ± 0.95 and 24.13 ± 1.01 for the right and left eyes, respectively.

A Pearson correlation test revealed significant correlation between MSE and AL for both the right and left eyes (*p* < 0.01 for both) indicating that any myopia was axial in nature. A paired samples *t* test showed no significant difference between eyes for axial length or MSE (*p* > 0.05 for both).

Mean values ± standard deviations for *k*, *k*′, *k*″, and *n* were 16.82 ± 6.35 (CI 12.98–20.66); 6.23 ± 1.44(CI 5.36–7.10); 273.77 ± 98.17(CI 214.44–333.10); and 2.16 ± 0.24 (CI 2.02–2.30), respectively.

### MSE

**Fig. 1:**
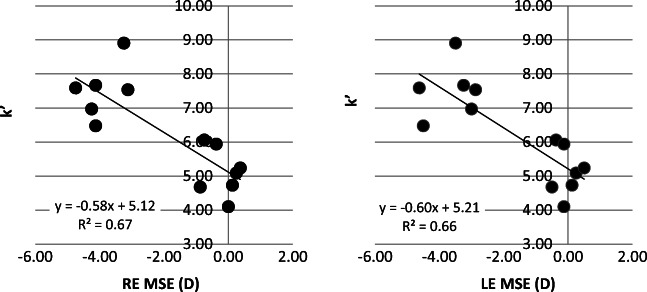
Changes in *k*′ with mean spherical error (MSE)

**Fig. 2:**
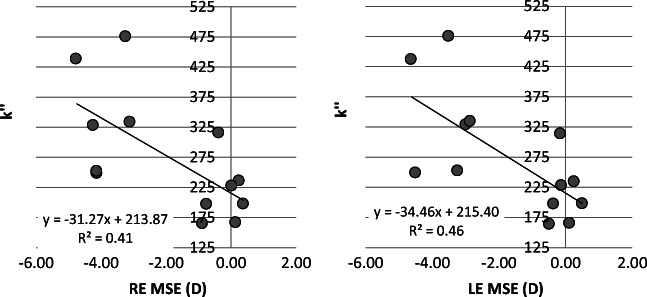
Changes in *k*″ with mean spherical error (MSE)

A significant correlation was found between increasing myopic MSE and increase in both the integral of light scatter (*k*′) (RE, *p* < 0.01; *r*^2^ 0.67; LE, *p* < 0.01; *r*^2^ = 0.66) and the total volume of light scatter (*k*″) (RE, *p* = 0.02; *r*^2^ 0.41; LE, *p* = 0.01, *r*^2^ = 0.46). Since the correlation between AL and MSE is significant, it remains uncertain as to whether the increased amount of light scattered in the eye can be attributed to increasing myopic MSE or simply to increased AL. When controlling for axial length as a covariate in the statistical analysis, the only remaining statistically significant correlation is with *k*′ (RE, *p* < 0.05; *r* = 0.64; LE, *p* < 0.05, *r* = 0.66) (see Figs. 1 and 2). There was no significant relationship found between the angular distribution of light scatter (parameter *n*) and refractive error (*p* > 0.05 for both eyes), or refractive error and the straylight parameter *k* (*p* > 0.05). Use of a quadratic (2nd order polynomial) fits for Fig. 1 (*k*′ vs. MSE) showed the *r*^2^ value improved to 0.72 for both eyes.

### Axial length

**Fig. 3:**
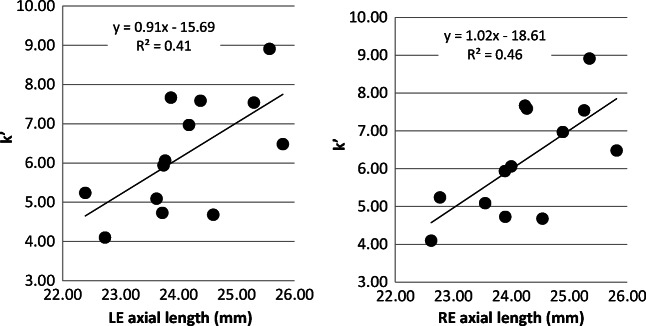
Changes in *k*′ with axial length

**Fig. 4:**
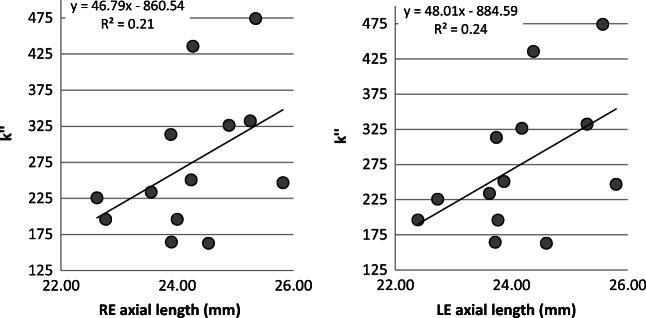
Changes in *k*″ with axial length (*p* > 0.05)

For axial length, significant correlations were noted between a longer axial length and increased in the integral of light scatter (*k*′) (RE, *p* = 0.01; *r*^2^ = 0.41; LE, *p* = 0.02, *r*^2^ = 0.46) (see Fig. 3); however, the effect disappeared for both the right and left eyes when controlling for MSE as a covariate (*p* > 0.05 for both eyes). This analysis is equivalent to that described for MSE. No significant relationships were found between n and axial length (*p* > 0.05 both eyes), or between axial length and the total volume of light scatter, *k*″ (see Fig. 4). There was also no significant relationship between axial length and the straylight parameter, *k* (*p* > 0.05 both eyes).

## Discussion

In general, the results are in agreement with the findings published by Rozema et al. [15]. Increase in myopia and the (inevitable) increase in the axial length of the eye were correlated with an increase in the total amount of light scatter in the eye. This is not surprising since either an increase in refractive error or the axial length of the eye may correlate with structural changes in the cornea or some other structure in the eye that causes the measured increase in the amount of scattered light. When the refractive error was controlled, axial length failed to show a good correlation with the amount of scatter light in myopic eyes. The results reported here are different than those reported by Guber et al. [16], who found emmetropes generally showed more light scatter than myopes. Our data also show that whilst refractive error affected significantly the amount of scattered light in the eye, in contrast, the angular distribution of scattered light as reflected in the straylight parameter, *n*, showed no significant correlation with either axial length or MSE.

Due to the paucity of work in this field, the source of variation in light scatter with respect to refractive error remains unclear. The reasons why the various studies that examined light scatter in myopes produced inconsistent results [15–18] also remain unexplained, although the use of different light scatter measuring apparatus and techniques may have played a part.

Based on what is known to cause forward light scatter in the eye, the results from this study suggest that myopic eyes may develop greater structural changes in either the cornea, the lens, or both when compared with emmetropic eyes. Participants with cataracts, history of refractive surgery, and corneal disease were excluded from the study. Any structural changes in the cornea and/or lens in the participants investigated are therefore likely to be physiological. Whilst it is known that there may be biomechanical changes to the cornea in myopia [22–24], there is little data about the corneal cellular structure with respect to either refractive error or axial length. That axial length failed to correlate with the measured light scatter parameters lends support to the view that the anterior eye, and not the retina, contributes most of the measured variation in scattered light.

A limitation of the current study could be that assessment of the light scatter function was undertaken binocularly. If, however, the two eyes differed significantly in their light scattering properties, the measurement of scattered light using the flicker-nulling test would have been very difficult since no single null point would apply to both eyes. The participants, however, had no difficulty in nulling the flicker caused by scattered light, even when the test was carried out binocularly. This observation suggests that the differences in light scatter between the two eyes must have been small. This is unsurprising, given the small anisometropic differences between the two eyes (i.e. ≤ 1.25 D). One can also argue that if the amount of scattered light measured relates directly to refractive error, the relationship between the mean (inter-eye) refractive error and the light scatter parameters should also be examined. In addition to examining the relationship between refractive error and the amount of scattered light for each eye separately, we also investigated what happens when the refractive errors were averaged. Each of the three analyses showed the same correlations, which suggest that the differences in light scatter parameters between the two eyes for each study participant were small.

One theoretical confounding factor in studies involving myopic participants may be the long-term use of contact lenses and associated corneal oedema. In our cohort, this is unlikely to be the case; modern contact lens materials generally offer high oxygen permeability, thus minimizing the likelihood of corneal oedema. Nevertheless, light scatter is also reported to increase in individuals with corneal surface disturbances caused by ‘dry eye’ or superficial punctuate keratitis [25] which are likely to be more prevalent amongst contact lens wearers and/or myopes [26]. Further, wearing a contact lens during the light scatter test can, in itself, adversely affect the results [13, 27]. Another potential source of bias may be the use of spectacles; if lenses were scratched or insufficiently cleaned, these factors may have contributed a small amount to the increased light scatter observed in myopes.

Pupil size may be affected by refractive status [28]. Although the measured light scatter parameters are independent of pupil size [29], this is only so when the light scattering is uniform over the pupil. Several reports have linked retinal straylight to pupil size, and these suggest that forward light scatter may not be uniform over the pupil [30–32].

In summary, the preliminary data from this study provide evidence that the total amount of scattered light in myopic eyes correlates with the magnitude of refractive error. Further studies are needed to characterize how changes in the anterior media of the eye, and a wider range of refractive errors, may affect forward light scatter.
